# Pío del Río Hortega and the discovery of the oligodendrocytes

**DOI:** 10.3389/fnana.2015.00092

**Published:** 2015-07-07

**Authors:** Fernando Pérez-Cerdá, María Victoria Sánchez-Gómez, Carlos Matute

**Affiliations:** Achucarro Basque Center for Neuroscience, Departamento de Neurociencias and CIBERNED, Universidad del País Vasco (UPV/EHU)Leioa, Spain

**Keywords:** Del Río Hortega, myelin sheath, oligodendroglia, oligodendrocyte precursor cell (OPC), Ramón y Cajal

## Abstract

Pío del Río Hortega (1882–1945) discovered microglia and oligodendrocytes (OLGs), and after Ramón y Cajal, was the most prominent figure of the Spanish school of neurology. He began his scientific career with Nicolás Achúcarro from whom he learned the use of metallic impregnation techniques suitable to study non-neuronal cells. Later on, he joined Cajal’s laboratory. and Subsequently, he created his own group, where he continued to develop other innovative modifications of silver staining methods that revolutionized the study of glial cells a century ago. He was also interested in neuropathology and became a leading authority on Central Nervous System (CNS) tumors. In parallel to this clinical activity, del Río Hortega rendered the first systematic description of a major polymorphism present in a subtype of macroglial cells that he named as oligodendroglia and later OLGs. He established their ectodermal origin and suggested that they built the myelin sheath of CNS axons, just as Schwann cells did in the periphery. Notably, he also suggested the trophic role of OLGs for neuronal functionality, an idea that has been substantiated in the last few years. Del Río Hortega became internationally recognized and established an important neurohistological school with outstanding pupils from Spain and abroad, which nearly disappeared after his exile due to the Spanish civil war. Yet, the difficulty of metal impregnation methods and their variability in results, delayed for some decades the confirmation of his great insights into oligodendrocyte biology until the development of electron microscopy and immunohistochemistry. This review aims at summarizing the pioneer and essential contributions of del Río Hortega to the current knowledge of oligodendrocyte structure and function, and to provide a hint of the scientific personality of this extraordinary and insufficiently recognized man.

## Biographical Sketch of Del Río Hortega

Pío del Río Hortega (1882–1945) was, with the exception of Ramón y Cajal, the most prominent figure of the Spanish school of neurology (Andres-Barquin, 2002; Pasik and Pasik, 2004; De Carlos and Pedraza, 2014). He revolutionized the study of neuroglia by developing and improving metallic impregnation techniques that he applied to the study of the group of non-astrocytic cells. These cells were poorly stained with the methods available at that time, and were known after Ramón y Cajal as the “third element” of Central Nervous System (CNS), neurons and astrocytes being the “first and second element”, respectively (Ramón y Cajal, 1913a). With the staining tools he developed, Del Río Hortega was able to identify two kinds of cells and to unveil their origin: microglia, the true “third element” due to its mesodermic origin; and oligodendroglia, included with astrocytes as second element due to their shared ectodermal origin (Del Río Hortega, 1918, 1920, 1924).

Pío del Río Hortega studied Medicine (1899–1905) and even as a student, he committed himself to follow a career in research which was focused on neurohistology and pathology all his professional life (exhaustively reviewed in Cano-Díaz, 1985; Del Río Hortega, 1986; López-Piñero, 1990). With some delay but with an enormous capacity for sustained hard work, he began his postdoctoral training in 1911, in Nicolás Achúcarro’s laboratory in Madrid (Spain), the year after. Achúcarro was Del Río Hortega’s true mentor and inculcated in him a deep interest in neuroglia before he worked in several European laboratories for short periods. After finally returning to Spain in 1914, he had the opportunity to share scientific interests with Ramón y Cajal, to whom he always felt great admiration, since Cajal’s and Achúcarro’s laboratories were located in the same building though each did independent research. Following closely in Achúcarro footsteps, and stimulated by Cajal’s third element, Del Río Hortega, now working full time in the laboratory, began to search for more stable variations of Cajal’s and Achúcarro’s metallic impregnation methods to study this cell class (reviewed in Castellano-López and González-de Mingo, 1995). In that fertile scientific environment, Del Río Hortega made numerous adjustments to the staining procedures which accounted for more than one hundred variations by the end of his career.

After developing modifications of Achúcarro’s ammoniacal silver method (Del Río Hortega, 1916), Del Río Hortega challenged the accuracy of Ramón y Cajal’s concept about the third element of CNS which grouped non neuronal (first element) and non-astrocytic (second element) cells (Ramón y Cajal, 1913b, 1916; García-Marín et al., 2007). Later, he described his silver carbonate staining technique which was the methodological key to identify two distinct elements: the microglia, the “true third element”, and what he called initially “interfascicular cells” and later oligodendroglia (Del Río Hortega, 1918, 1920, 1921). Ramón y Cajal and others were not convinced particularly regarding the existence of oligodendroglia (reviewed in Pasik and Pasik, 2004). Perhaps, this skepticism delayed the immediate acceptance of these cells, and contributed to a misunderstanding between the two scientists which ended up with the dismissal of Del Río Hortega from Cajal’s laboratory in 1920 and his move to a new one, promoted in some ways by Ramón y Cajal himself. He was aware of Del Río Hortega talent as researcher and although they never worked again together, their relationship improved later on.

Once in his own laboratory, Del Río Hortega continued frantically with his investigations and created an important school with outstanding pupils from Spain and abroad. Among them was Penfield, who greatly supported and replicated the results of Del Río Hortega, and thus, contributed to the international recognition of Del Río Hortega’s discovery of oligodendroglia (Penfield, 1924; Gill and Binder, 2007). This intense activity was favored by the commitment of the Spanish Government of that time to guarantee high standards in science, an atmosphere that helped Del Río Hortega to develop a well equipped Laboratory of Histology and Pathology, as he named it (Andres-Barquin, 2002; De Carlos and Pedraza, 2014). At the same time, Del Río Hortega himself was an active advocate of science both within and outside of the academic circles. Del Río Hortega became internationally recognized for his contributions to the understanding of glia in the healthy nervous system and also in disease, mainly in cerebral tumors. Unfortunately, the Spanish civil war (1936–1939) forced him into exile which interrupted the development of his school, though he strived to keep it alive in the midst of difficulties while working abroad in Oxford and Buenos Aires (reviewed in Cano-Díaz, 1985; López-Piñero, 1990).

## Silver Carbonate Staining Method of Del Río Hortega

All along his career, del Río Hortega had a great interest in improving metallic impregnation techniques to advance the characterization of neural cells (reviewed in Castellano-López and González-de Mingo, 1995; Pasik and Pasik, 2004). He developed new modifications to the Achúcarro’s ammoniacal silver staining (Del Río Hortega, 1916), applied Cajal’s formol uranium nitrate and gold chloride sublimate methods (Ramón y Cajal, 1913b, 1916), as well as the Golgi’s method. This array of techniques gave him and those who used them, an almost complete picture of the morphology of the protoplasmic and fibrous astrocytes (FAs), cells known as the second element of the CNS, neurons being the first element. However, these methods did not stain the remaining cell types of the CNS which were termed by Ramón y Cajal as the third element which in his own words was composed solely of “corpuscles without processes” grouping adendritic, apolar dwarf cells that were present in white matter, perineuronally and as perivascular satellites (Ramón y Cajal, 1913a, 1916; García-Marín et al., 2007).

The identification of these cells was possible when Del Río Hortega described a method of using silver carbonate to stain glial cells (Del Río Hortega, 1918) with precise timing of the formalin-ammonium bromide fixative introduced by Ramón y Cajal (1913b). Del Río Hortega never explained how (i.e., a mistake, an intuition, or a test) he happened to introduce lithium carbonate with silver nitrate to precipitate it as silver carbonate (Del Río Hortega, 1918), but it could be said that in the best Cajalian tradition, he doggedly tried modification after modification of methods to selectively stain cell types. His discovery provided Del Río Hortega with a new tool to transform morphological and physiological concepts of the CNS. For the first time, this method clearly distinguished two cells types with distinct cytoplasmic expansions in the previously so-called third element group, which Del Río Hortega termed microglia and oligodendroglia (Del Río Hortega, 1920, 1921). He focused his research efforts on microglia and found its mesodermal origin (the true third element), its surveillance function and phagocytic capacity in pathology in a remarkably precise fashion, which was soon accepted by the scientific community. However, there was still much debate on the existence of oligodendroglia as a distinct CNS cell type, particularly by Ramón y Cajal and others (reviewed in Pasik and Pasik, 2004). It was not until 1924 when the confirmation of oligodendroglia as a variety of neuroglia of ectodermic origin (part of second element as astroglia was) was broadly accepted (Del Río Hortega, 1924; Penfield, 1924; Gill and Binder, 2007).

## Contribution of Del Río Hortega to Understanding Oligodendroglia

**Figure 1:**
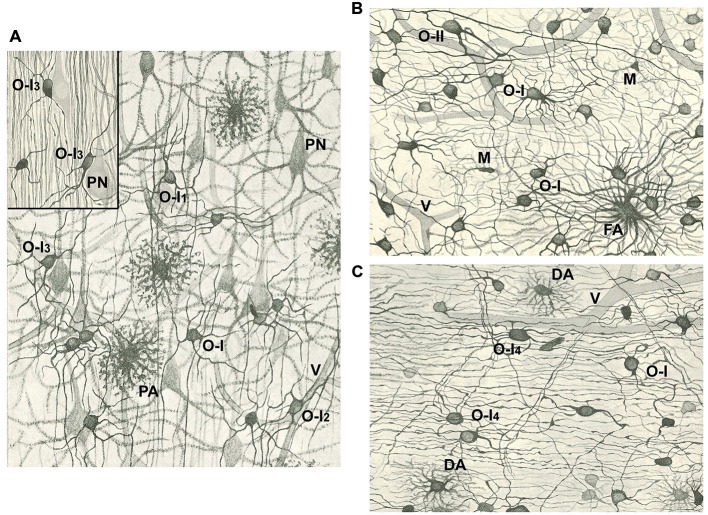
**Drawings of the cerebral cortex (A) and white matter (B,C) after staining with the Golgi-Hortega method or the silver carbonate procedure by Hortega (inset in A). (A)** Notice pyramidal neurons (PN), protoplasmic astrocytes (PA), vessels (V), and type I oligodendrocytes (OLGs; O-I) with variable number of processes, many of them divided in “Y” or “T”. Some OLGs have processes mainly oriented in the direction of projecting axons (O-I_1_), while others have a perivascular (O-I_2_) or perineuronal (O-I_3_; see inset) localization. **(B)** Note a fibrous astrocyte (FA), some OLGs of the first type (O-I) and one of the second type (O-II), as well as microglia cells (M). **(C)** See type I OLGs similar to those in **(A, B)** (O-I) or with long processes that follow axons (O-I_4_), and two dwarf astrocytes (DA). Vessels (V) are also drawn in **(B,C)**. Magnification in Figures **(A–C)** is similar. Modified from Del Río Hortega (1928).

**Figure 2:**
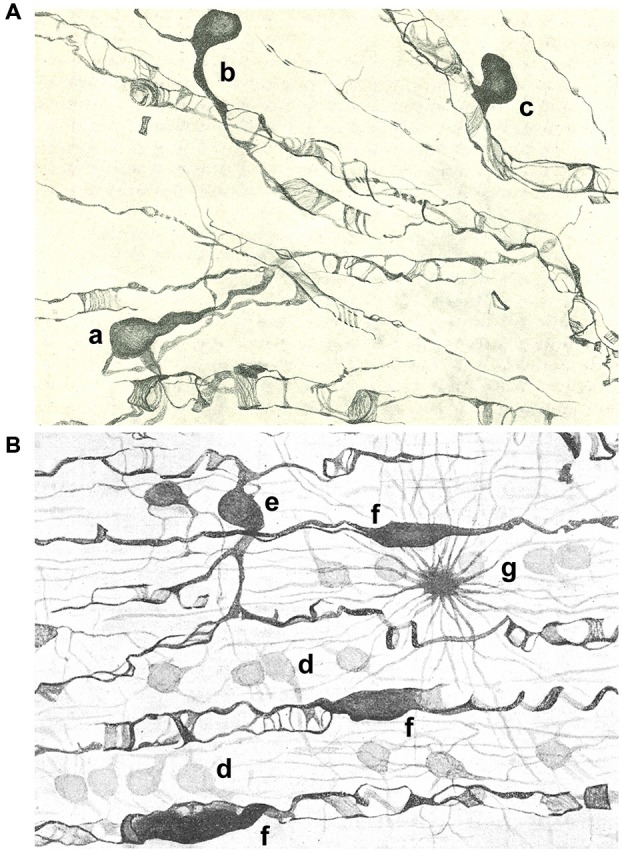
**Drawings of the subcortical (A) and spinal cord (B) white matter after staining with the Golgi-Hortega method. (A)** Display of oligodendrocytes of the third type with different kind of processes around axons are represented: one has two clear and long processes (a), while others are endowed with a single process divided into an acute angle for two axons (b) or in T that ensheathes a nerve fiber (c). **(B)** Illustration of oligodendrocytes of the first (d); third [e; similar to a in panel **(A)**] and fourth type (f) as well as a fibrous astrocyte (g). Modified from Del Río Hortega (1928).

Del Río Hortega rendered the first systematic description of oligodendrocytes (OLGs) in an article published in 1928 (Del Río Hortega, 1928; Figures 1, 2). Nevertheless, the complete story of his discovery had already begun when he described microglia (Del Río Hortega, 1920; Castellano-López and González-de Mingo, 1995; Pasik and Pasik, 2004) as the third element, mentioning the existence of a new cell type of neuroglia, the interfascicular glia, made up by cells showing very fine processes and arranged in groups among axonal tracts. Surely this distinction was only made possible using the new silver carbonate impregnation method developed by him (Del Río Hortega, 1918). In 1921, he named these cells as oligodendroglia or glia with very few processes (Del Río-Hortega, 1921), because they were present not only in white matter but diffusely distributed in all regions of the CNS and commonly grouped next to neurons in gray matter. He was aware that as many other histochemical techniques involving metallic silver impregnations, his silver carbonate method had very specific requirements, which did not, however, guarantee reproducible results in every preparation. Despite the results were very variable in terms of staining, he predicted the relationship of oligodendroglia with myelination, its implication in neuronal trophism, and its ectodermal origin. In fact, one year later (Del Río Hortega, 1922) proposed that these cells were functionally similar to Schwann cells in the CNS and responsible for myelination. However, the demonstration of oligodendroglia as cells that produce and maintain the myelin sheaths that insulate CNS axons had to wait for the introduction of electron microscopy in the 1960s (reviewed in Verkhratsky and Butt, 2007; Butt, 2013). This temporal gap, together with difficulties in oligodendroglia staining until the introduction of immunohistochemical techniques, and that the seminal articles by Del Río Hortega were published in Spanish, made his discovery of oligodendroglia not recognized internationally, as his discovery of microglia was, and restricted to scientists, who were histologists (Castellano-López and González-de Mingo, 1995; Pasik and Pasik, 2004).

Del Río Hortega published a thorough review of his discoveries about morphology and functionality of oligodendroglia in 1928 (Del Río Hortega, 1928). By this time he had introduced a new metal impregnation protocol based on the Golgi method, known as Golgi-Hortega technique, which provided detailed information on the morphology of these cells, which he renamed as OLGs. He noted three kinds of OLGs according to their neighboring relationship: interfascicullar (alignment of closely apposed cells in rows along axonal tracts); perineuronal (juxtaposing neuronal soma) and perivascular (abutting blood vessels but lacking contacts; Figures 1, 2). He was astonished with the complexity of oligodendrocytic morphology which he profusely illustrated with drawings and photomicrographs in a review (Del Río Hortega, 1928). Accordingly, he tried to classify OLGs according to their soma size and shape, number and characteristics (orientation) of cellular processes, their distribution within CNS, manner of interaction with axons and size of the axons with which they were associated. As a consequence of this analysis, he grouped OLGs into four subtypes (I to IV), while recognizing the absence of clear boundaries among them.

Type I OLGs or Robertson’s OLGs, are named so because this type was probably the only one observed by Robertson (Robertson, 1899), have small rounded cell body (15–20 μm diameter) and a high number (from 5 to 20 or more) of very fine processes emerging in multiple directions and towards axons that are usually thinly myelinated. They are present in gray (nearly all perineuronal OLGs are of the first type) and white matter (frequently arranged in interfascicular series; Figures 1, 2).

Type II OLGs or Cajal’s OLGs, named as a tribute to him, are only present in white matter. They are polygonal or cuboidal in shape (20–40 μm) with fewer and thicker processes than type I OLGs, which are directed to axons and attached to them longitudinally (Figure 1B).

Type III OLGs or Paladino’s OLGs because Paladino, although associated with many misinterpretations, had intuited that myelin had a neuroglial origin (Paladino, 1892). Theseare also less abundant than types I and II. They are present in white matter with thick myelinated fibers (as brain stem and spinal cord) and are distinguished by one to four processes emanating from a bulky cell body and directed toward axons (Figure 2).

Type IV OLGs or Schwannoid OLGs, due to their similarity in appearance, are very elongated cells with flattened somata, and found adhered and extended mono or bipolarly to medium or large thickness axons in white matter of brainstem and spinal cord (Figure 2B).

This classification was not made for purely descriptive purposes. In fact, he also made a synthesis about the morphological and physiological knowledge of OLGs creating the concept of neurogliona (Del Río Hortega, 1942), by suggesting that OLGs have a close association with neurons and attributing to them hypothetically mechanical, trophic and myelinogenic functions. Although many observations along his scientific career supported the formation of myelin by OLGs, either directly or by supplying axons with needed materials, he was cautious enough not to consider them as definitive. This conclusion could be regarded as an example reflecting his high standards of scientific reasoning and intuition (Cano-Díaz, 1985; López-Piñero, 1990; Castellano-López and González-de Mingo, 1995; Andres-Barquin, 2002; Pasik and Pasik, 2004; Gill and Binder, 2007).

## Scientific Legacy of Del Río Hortega on Oligodendrocyte Knowledge

The oligodendrocyte phenotypic diversity proposed by Del Río Hortega was initially neglected, but it has been later on confirmed by electron microscopy, intracellular dye injection, immunohistochemistry and more recently with genetic tools (Butt, 2013). This could be due to the fact that his studies were made mainly in gyrencephalic brains while current consensus about OLGs has been mainly obtained from lysencephalic ones. Indeed, Del Río Hortega’s contribution to the field has been often overlooked and reference to his pioneer ideas are not included in recent relevant papers (for example: Nishiyama et al., 2014; Dumas et al., 2015; Zeisel et al., 2015). This oblivion is unfair since we learned from his discoveries that OLG phenotypes are related to the number of axons myelinated per OLG and the diameters of fibers they myelinate. As a result of that finding, we now classify OLGs in two distinct phenotypes defined by the caliber of the axon they myelinate, i.e., below and above of 2–4 μm of diameter which correspond to Del Río Hortega’s types I/II and III/IV (Verkhratsky and Butt, 2007; Butt, 2013). In addition, although he did not specifically mention it, he did suggest that there was a direct relationship between the axon caliber and the internodal length (i.e., the length between two nodes of Ranvier, the unmyelinated axonal gap where action potentials are generated), as well as with the width of the myelin sheath.

As of today, it is not clear how OLG polymorphism impacts the thickness and width of the myelin sheath and the functioning of the myelinated axons. In addition, recent evidence about axonal metabolic support provided by OLGs (Morrison et al., 2013; Saab et al., 2013) could be related to the concept of neurogliona suggested by Del Río Hortega (1942). It is outstanding that, as with Ramón y Cajal, he related morphology to function usingmicroscopy and neurohistological preparations impregnated with innovative and specific staining methods exclusively. This reveals an enormous capacity for hard work, deep observational abilities and exceptional artistic skills.

Current data show a population of adult oligodendrocyte progenitor cells, called NG2-glia or polydendrocytes, which provide a pool of slowly proliferating cells that generate OLGs throughout life (Nishiyama, 2013; Nishiyama et al., 2014). Del Río Hortega already observed this cell population in white matter (Del Río Hortega, 1928). He described it as a cell type with ambiguous character sharing with OLGs the size and shape of soma, but differing from them by the number and characteristics of its expansions: very numerous, not very long, dichotomized at acute angles several times with a semiprotoplasmic appearance similar to that of astrocytes which display a crown-like shape though its diameter is much smaller. He named them as dwarf astrocytes (DA) and although he did not propose a particular biological significance for those cells, they could possibly correspond to polydendrocytes, whose morphological descriptions are very similar (See Figure 1C). We now know that OLGs are not the only fate of polydendrocytes, particularly during development since they can differentiate into astrocytes (Nishiyama, 2013; Nishiyama et al., 2014).

Another exciting OLG type described by Del Río Hortega was the perineuronal one whose soma lie apposed to neuronal soma (Del Río Hortega, 1928). They are non-myelinating cells and although their role is not clear, they could provide neurotrophic and metabolic support for neurons as he suggested, an idea that others extended to pathology showing that they could produce myelin in response to demyelination (Nishiyama et al., 2014).

Del Río Hortega observations and interpretations have been instrumental to contemporary neurobiology. He anticipated concepts that were dormant during decades, due in part to the neurocentric view of the CNS, and of the view that astrocytes are the relevant glial cells in the understanding of physiology of CNS and its pathology. More recently, the interest in OLGs has had a renaissance with the increasing attention to translational research on demyelinating diseases, and ultimately, provide justice to the pioneer contributions to our knowledge of oligodendroglia made possible by Del Río Hortega. It would be difficult to imagine a coherent story of OLGs without recognizing his contributions.

## Molecular Epilog

Historically, OLGs have been classified using location and morphology, as started by Del Río Hortega (1928), in combination afterwards with molecular markers (reviewed in Butt, 2013). Although the majority of OLGs in any one category tend to look alike (see Figures 1, 2), very recently the analysis of the RNAs expressed in these brain cells (Zeisel et al., 2015) has revealed the possibility of classifying OLGs into a half-dozen classes according to progressive changes in previously known and novel gene expression markers along OLG differentiation. The harmonization of morphological and genetic criteria to classify OLGs remains to be done, and reveals the complexity of oligodendroglia. All in all, this open question reveals that the knowledge of the OLG network organization, pioneered by Del Río Hortega almost a century ago, is still an open question which needs further exploration.

## Conflict of Interest Statement

The authors declare that the research was conducted in the absence of any commercial or financial relationships that could be construed as a potential conflict of interest.
